# Epigenetic landscape of drug responses revealed through large-scale ChIP-seq data analyses

**DOI:** 10.1186/s12859-022-04571-8

**Published:** 2022-01-24

**Authors:** Zhaonan Zou, Michio Iwata, Yoshihiro Yamanishi, Shinya Oki

**Affiliations:** 1 Department of Drug Discovery Medicine, Kyoto University Graduate School of Medicine, 53 Shogoin Kawahara-cho, Sakyo-ku, Kyoto, 606-8507 Japan; 2 Kyoto University Graduate Program for Medical Innovation, Yoshida-Konoe-cho, Sakyo-ku, Kyoto, 606-8501 Japan; 3 Kyoto University Graduate Division, Yoshida-Nihonmatsu-cho, Sakyo-ku, Kyoto, 606-8501 Japan; 4 Department of Bioscience and Bioinformatics, Faculty of Computer Science and Systems Engineering, Kyushu Institute of Technology, 680-4 Kawazu, Iizuka, Fukuoka 820-8502 Japan; 5 Precursory Research for Embryonic Science and Technology, Japan Science and Technology Agency, 4-1-8 Honcho, Kawaguchi, Saitama 332-0012 Japan

**Keywords:** Drug modes of action, Transcriptome, ChIP-seq, Transcription factor, Epigenetic landscape

## Abstract

**Background:**

Elucidating the modes of action (MoAs) of drugs and drug candidate compounds is critical for guiding translation from drug discovery to clinical application. Despite the development of several data-driven approaches for predicting chemical–disease associations, the molecular cues that organize the epigenetic landscape of drug responses remain poorly understood.

**Results:**

With the use of a computational method, we attempted to elucidate the epigenetic landscape of drug responses, in terms of transcription factors (TFs), through large-scale ChIP-seq data analyses. In the algorithm, we systematically identified TFs that regulate the expression of chemically induced genes by integrating transcriptome data from chemical induction experiments and almost all publicly available ChIP-seq data (consisting of 13,558 experiments). By relating the resultant chemical–TF associations to a repository of associated proteins for a wide range of diseases, we made a comprehensive prediction of chemical–TF–disease associations, which could then be used to account for drug MoAs. Using this approach, we predicted that: (1) cisplatin promotes the anti-tumor activity of TP53 family members but suppresses the cancer-inducing function of MYCs; (2) inhibition of RELA and E2F1 is pivotal for leflunomide to exhibit antiproliferative activity; and (3) CHD8 mediates valproic acid-induced autism.

**Conclusions:**

Our proposed approach has the potential to elucidate the MoAs for both approved drugs and candidate compounds from an epigenetic perspective, thereby revealing new therapeutic targets, and to guide the discovery of unexpected therapeutic effects, side effects, and novel targets and actions.

**Supplementary Information:**

The online version contains supplementary material available at 10.1186/s12859-022-04571-8.

## Introduction

Elucidating the modes of action (MoAs) of drugs and candidate compounds is critical for guiding translation from drug discovery to clinical application. Understanding the complex responses of the human biological system to chemicals is of vital importance in medical and pharmaceutical research. For many chemicals, including some approved drugs, the MoAs remain elusive. The task of revealing MoAs can be moderately simplified to the identification of target proteins implicated in the pharmacological effects of chemicals on disease. Phenotype-based high-throughput screening (PHTS) is an efficient way to find candidate compounds with a desired phenotype [1–3]. Although PHTS can rapidly screen thousands of chemicals, the underlying molecular mechanisms of the hit compounds remain unknown. Identification of the target proteins associated with a phenotype requires considerable effort, e.g., analysis of drug–protein interactions using biochemical and chemoinformatic methods [4–11]. Recent developments in biotechnology have contributed to the increase in the amounts of omics data for chemicals and proteins in the genome, transcriptome, epigenome, and interactome, which can be useful sources for inferring the MoAs of drugs.

Based on the concept that gene expression changes are pivotal for pharmacophysiological effects, another major approach to drug discovery is transcriptome profiling following administration of compounds. A popular transcriptome-guided drug repositioning approach for finding novel drugs is to search for compounds whose gene expression patterns are inversely correlated with those of a disease of interest [12–16]. These methods involve genome-wide expression profiling of transcriptional responses to compound treatment. In recent years, chemically induced gene expression data based on large-scale transcriptome experiments have been made available from several public databases. The Toxicogenomics Project-Genomics Assisted Toxicity Evaluation system (TG-GATEs) hosts the results of a toxicogenomics project implemented in Japan, in which 170 compounds were used to perturb cell homeostasis in vitro [17]. Subsequently, the Connectivity Map (CMap) database was constructed to provide the gene expression profiles of five cancer cell lines perturbed by 1,309 compounds [18]. Many more compounds have been examined by the National Institute of Health Library of Integrated Network-Based Cellular Signatures (LINCS) consortium, which analyzed the transcriptomic responses of 68 human cell lines to more than 20,000 compounds [19]. The LINCS consortium took advantage of a “reduced representation of the transcriptome”, in which 978 landmark genes, termed L1000, are investigated as a representative gene set for biological significance. The Comparative Toxicogenomics Database (CTD) is another public resource that provides information about differentially expressed genes (DEGs) following administration of chemicals and medical drugs [20]. The distinguishing feature of CTD is that all records were generated by manual curation of more than 13,713 peer-reviewed publications, including expression changes of 23,081 genes upon administration of 4,121 compounds to human cells. Thus, relative to the aforementioned large-scale projects, CTD integrates a wider variety of genes in an unbiased manner.

Despite the construction of chemically induced gene expression signatures, the molecular cues that mediate gene expression changes in response to chemical administration remain to be clarified. One class of promising mediators are the transcription factors (TFs) that act upstream of sets of chemically induced DEGs to control their expression. TFs are master regulators that profoundly alter cell phenotype and behavior by modulating the epigenetic landscape, thereby organizing the expression of large sets of genes. Although it is true that TFs are not often directly targeted by drugs, recent studies have demonstrated the power of epigenetic drug discovery, e.g., by revealing the potential utility of inhibitors of bromodomain proteins and histone deacetylases (HDACs) against neoplasms [21–25]. These findings highlight the importance of modeling epigenetic landscapes in the context of pharmacological strategies. Although specifically targeting TF activity faces major hurdles, targeting effectors downstream of cell signaling (e.g., TFs) rather than upstream factors is likely to be a more specific approach [26]. In this study, instead of predicting directly druggable targets, we developed a computational method for identifying TFs pivotal to thousands of chemically induced DEGs, making full use of large-scale TF binding profiles obtained from tens of thousands of actual chromatin immunoprecipitation sequencing (ChIP-seq) datasets. The predicted chemical–TF associations provided clues about drug MoAs involved in drug efficacies and side effects. Our approach outperformed methods that directly evaluated the similarity of chemically induced and disease-specific DEGs without considering key TFs.

## Results

### Overview of TF-focused elucidation of drug MoAs

**Fig. 1:**
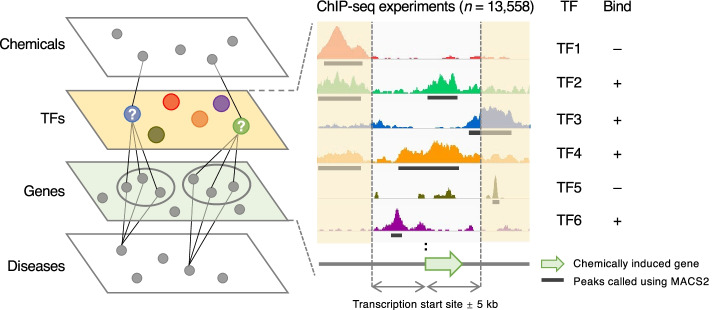
Overview of the proposed ChIPEA-based approach. To elucidate the epigenetic landscape of drug responses, we identified the TFs enriched on chemically induced genes by analyzing large-scale ChIP-seq data. Overlaps were evaluated between the transcription start site ± 5 kb regions of chemically induced genes (green arrow) and peak-call data (black lines) of 13,558 TF-related experiments archived in ChIP-Atlas

A considerable proportion of bioactive compounds and medical drugs exert their effects by modifying disease-elicited gene expression. To further understand the MoAs of chemicals and, in turn, to define chemical–disease associations, it is of crucial importance to focus on the master regulators that organize the expression of chemically perturbed DEGs. In the proposed approach, shown in Fig. 1, we identified TFs that integratively regulate chemically perturbed DEGs by analyzing large-scale comprehensive ChIP-seq data obtained from ChIP-Atlas [27]. In addition, we evaluated the matches of predicted target TFs with disease-associated proteins available from the DisGeNET database [28] (details shown below). The predicted chemical–TF and chemical–disease associations were validated with known chemical–protein associations from the Kyoto Encyclopedia of Genes and Genomes DRUG database (KEGG DRUG) [29] and the chemical–disease association dataset from CTD, respectively.

### Evaluation of biological significance of genes and TFs in databases

To confirm the quality of chemically induced transcriptomes, we evaluated the biological significance of annotated chemically perturbed genes by comparing the statistics of gene expression profile data in CTD and L1000 in order to estimate their overlap. Additional file 1: Figure S1a shows a Venn diagram of the genes that overlapped among the comparisons. Almost all (99.3%) of the L1000 landmark genes are also included in CTD. We sorted chemically induced genes in the order of the frequency with which they appeared in CTD, and assessed their matches with L1000 landmark genes (Additional file 1: Fig. S1b). The results confirmed that the representative L1000 genes were ranked generally higher among the genes annotated in CTD (*p* = 2.5 × 10^−176^ by Wilcoxon rank-sum test). In particular, 41 DEGs in the top 100 of CTD were designated as L1000 genes. These data suggest that CTD includes a wide variety of biologically significant genes.

We then compared the annotated proteins in DisGeNET and ChIP-Atlas (Additional file 1: Fig. S1c). Most of the TFs profiled by ChIP-seq experiments (67.9% of ChIP-Atlas TFs) are also curated in DisGeNET. We sorted disease-associated proteins in the order of the frequency with which they appeared in DisGeNET, and assessed the match with TFs contained in ChIP-Atlas (Additional file 1: Fig. S1d). The results confirmed that the TFs were ranked generally higher among the proteins annotated in DisGeNET (*p* = 3.2 × 10^−33^ by Wilcoxon rank-sum test). These data suggest that the TFs analyzed by ChIP-seq associate with diseases more strongly than other proteins within DisGeNET. In summary, CTD, ChIP-Atlas, and DisGeNET include information about biologically significant genes and proteins, and are therefore suitable for elucidating the MoAs of chemicals and inferring chemical–disease associations.

### Identification of master regulators that organize the expression of DEGs in response to drug treatment

Chemical perturbation of gene expression is organized by a series of TFs in an integrated manner. Therefore, identification of key TFs is critical for understanding drug MoAs. To this end, we combined chemically induced DEGs from CTD (Additional file 2: Table S1) and large-scale public ChIP-seq data (*n* = 13,558) from ChIP-Atlas (Fig. 1, Additional file 2: Table S2). We then performed ChIP-seq-based enrichment analysis (ChIPEA, detailed in Methods) to identify TFs that exhibited enriched binding around up- or down-regulated genes following drug administration (target range: transcription start site ± 5 kb).

We then asked whether the TFs with higher enrichment scores were involved in the MoAs of query compounds. As standard data, we used known chemical–protein interactions data obtained from KEGG DRUG and applied the receiver operating characteristic (ROC) curve, a plot of true-positive rates as a function of false-positive rates, as well as the precision-recall (PR) curve, which is a plot of precision (positive predictive value) as a function of recall (sensitivity). We then summarized the evaluation using the area under the ROC curve (AUROC) score, where 1 is perfect classification and 0.5 is random classification, and the area under the PR curve (AUPR) score, where 1 is perfect inference and the ratio of positive examples in the standard data is random inference.

**Fig. 2:**
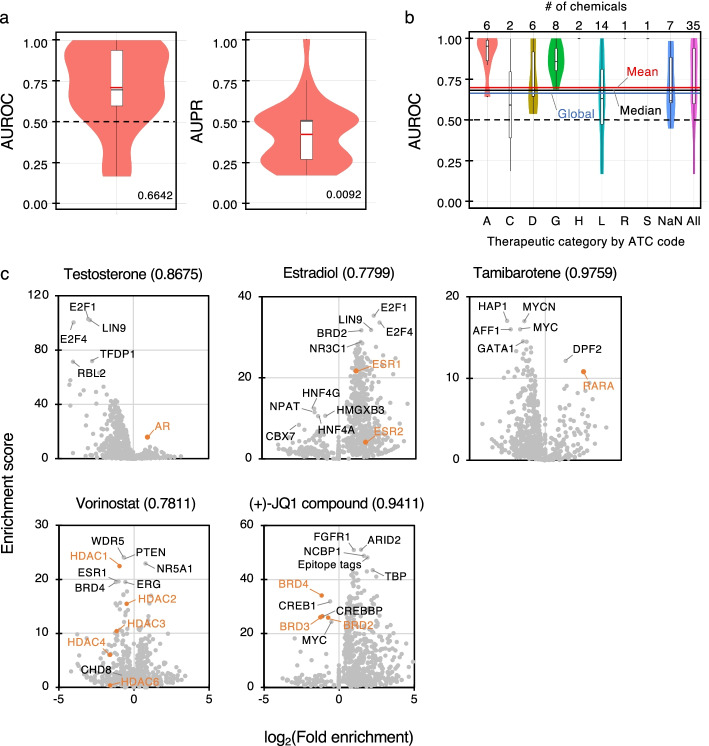
Identification of target TFs by ChIPEA.** a** Distribution of AUROC and AUPR scores for each chemical–TF association predicted using ChIPEA. Red and black horizontal lines inside the box represent mean and median scores, respectively, and global scores are noted beside the violin plots. **b** Distribution of AUROC scores by chemical class according to the first level of the Anatomical Therapeutic Chemical classification system (ATC code). Chemicals are assigned the following ATC codes. A: alimentary tract and metabolism; B: blood and blood-forming organs; C: cardiovascular system; D: dermatologicals; G: genitourinary system and sex hormones; H: systemic hormonal preparations, excluding sex hormones and insulins; J: anti-infectives for systemic use; L: antineoplastic and immunomodulating agents; M: musculoskeletal system; N: nervous system; P: anti-parasitic products, insecticides and repellents; R: respiratory system; S: sensory organs; V: various; NaN: not assigned. The numbers of chemicals assigned to each ATC code are noted above the violin plots. Mean, median, and global AUROC scores are shown with red, black, and blue horizontal lines, respectively. **c** Predicted target TFs of five representative chemicals. Dots indicate individual TFs and are colored orange if they matched chemical–target associations recorded in KEGG DRUG. AUROC scores indicating the accuracy of the chemical–TF association inference are shown in parentheses following the names of the chemicals. Enrichment scores (−log_10_[*p*-value]) were calculated using the two-tailed Fisher’s exact test. The null hypothesis is that the intersection of the reference peaks for up-regulated genes occurs in the same proportion as for those with down-regulated genes. Fold enrichment (detailed in Methods) was calculated using the same ChIP-seq data and the following equation: (overlaps/up-regulated genes)/(overlaps/down-regulated genes)

The distribution of AUROC and AUPR scores for each chemical (*n* = 35) that directly targets TFs was visualized with a violin plot (Fig. 2a). Mean AUROC and mean AUPR across chemicals were 0.7063 and 0.4187, respectively. Given that the distribution pattern of AUROC varied depending on the highest enrichment score of each chemical (Additional file 1: Fig. S2), we were concerned that the item discrimination to distinguish associated and not associated TFs within individual chemicals was in some sense limited. Therefore, we calculated “global” statistics using an inter-chemical merged enrichment score vector to emphasize the significance of the actual values of enrichment scores (detailed in the Methods section). The global AUROC and global AUPR scores were 0.6642 and 0.0092, respectively. Figure 2b shows the distribution of AUROC scores for a number of chemical classes on the basis of the first level of the WHO Anatomical Therapeutic Chemical (ATC) Classification code; the detailed explanations on the ATC codes are shown in the figure caption. These results revealed that ChIPEA can generally predict chemical–TF associations with high efficiency, particularly for chemicals categorized as ATC code A (alimentary tract and metabolism, e.g., diabetes treatments and vitamins) and G (genitourinary system and sex hormones), which directly target nuclear receptors.

Figure 2c illustrates representative predictions of chemical–TF associations (Additional file 2: Table S3). For example, androgen receptor (AR), estrogen receptor (ESR) 1/2, and retinoic acid receptor alpha (RARA) were shown to significantly bind to the up-regulated genes induced by testosterone (primary male sex hormone), estradiol (major female sex hormone), and tamibarotene (synthetic retinoid) treatment, respectively [30–32]. Though they did not exhibit the highest enrichment, the binding of histone deacetylases (HDACs) was clearly detected among the TFs that bound in a biased manner to down-regulated genes after treatment with vorinostat, a pan-HDAC inhibitor. In addition, bromodomain-containing proteins (BRDs), which are crucial factors involved in the transcription elongation process, were enriched among the down-regulated genes after treatment with (+)-JQ1 compound (JQ1), a bromodomain and extra-terminal motif (BET) protein inhibitor and potential antineoplastic agent. These results suggest that testosterone, estradiol, and tamibarotene promote, whereas vorinostat and JQ1 suppress, the activities of the corresponding receptors or factors in a manner consistent with the evidence [30–34].

### Highlighting MoAs along with presumed chemical–TF–disease associations of CTD chemicals

To examine the MoAs more deeply, and in turn construct the chemical–TF–disease associations, we linked the TFs enriched for chemically induced DEGs by ChIPEA with protein–disease associations according to the DisGeNET database. In the proposed method, the probabilities of chemical–TF–disease associations were simply represented by the ChIPEA enrichment scores. For comparison, we also used a conventional DEG-connected method for chemical–disease association analysis, which directly calculates the positive or negative correlation between chemically induced DEGs from CTD and disease-specific DEGs from Crowd Extracted Expression of Differential Signatures (CREEDS) [35]. The sets of genes included in CTD and CREEDS modestly overlapped with each other (69.3% and 81.1% of genes, respectively), and CREEDS was confirmed to include an extensive range of biologically significant genes suitable for inferring chemical–disease associations (Additional file 1: Fig. S1a, b).

**Fig. 3:**
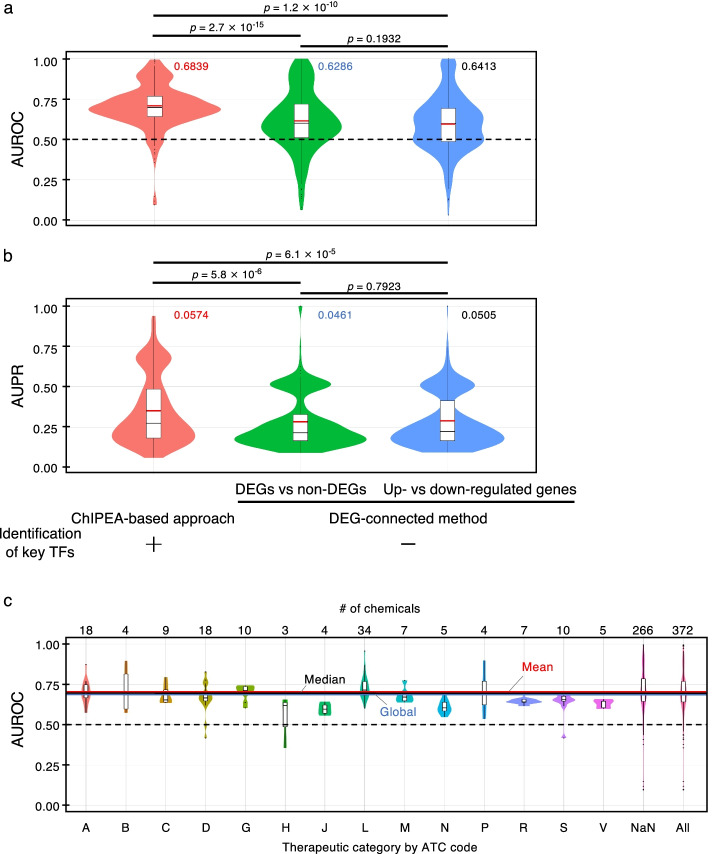
Prediction of chemical–TF–disease associations. **a, b** Distributions of AUROC (**a**) and AUPR scores (**b**) for each chemical–disease association predicted using the proposed ChIPEA-based approach and approaches that ignore TFs. Red and black horizontal lines inside the box represent mean and median scores, respectively, and global scores are noted beside the violin plots. Differences between the methods are presented with *p*-values by Wilcoxon rank-sum test above the violin plots. **c** Distribution of AUROC scores by chemical class according to the first level of the Anatomical Therapeutic Chemical classification system (ATC code). Chemicals are assigned the following ATC codes. A: alimentary tract and metabolism; B: blood and blood-forming organs; C: cardiovascular system; D: dermatologicals; G: genitourinary system and sex hormones; H: systemic hormonal preparations, excluding sex hormones and insulins; J: anti-infectives for systemic use; L: antineoplastic and immunomodulating agents; M: musculoskeletal system; N: nervous system; P: anti-parasitic products, insecticides and repellents; R: respiratory system; S: sensory organs; V: various; NaN: not assigned. The numbers of chemicals assigned each ATC code are noted above the violin plots. Mean, median, and global AUROC scores are shown with red, black, and blue horizontal lines, respectively

To compare the accuracies of predicted chemical–disease associations between the proposed method and the DEG-connected method, we applied ROC and PR curves using known chemical–disease associations obtained from CTD as standard data. The global AUROC and global AUPR of the proposed approach were 0.6839 and 0.0574, respectively (mean AUROC = 0.7026, mean AUPR = 0.3504), higher than that of approaches that only compared DEGs versus non-DEGs (global AUROC = 0.6286, mean AUROC = 0.6133, global AUPR = 0.0461, mean AUPR = 0.2790; *p* = 2.7 × 10^−15^ for AUROC, *p* = 5.8 × 10^−6^ for AUPR by Wilcoxon rank-sum test) or up- versus down-regulated genes (global AUROC = 0.6413, mean AUROC = 0.5972, global AUPR = 0.0505, mean AUPR = 0.2846; *p* = 1.2 × 10^−10^ for AUROC, *p* = 6.1 × 10^−5^ for AUPR by Wilcoxon rank-sum test) (Fig. 3a). Figure 3b shows the distribution of AUROC scores of the purposed method, stratified by ATC code. These results suggest that the identification of target TFs using ChIPEA is a powerful approach that can be used to clarify pivotal factors for specific MoAs and is therefore useful for predicting the diseases associated with treatments, particularly for chemicals categorized as ATC code G (genitourinary system and sex hormones) or L (antineoplastic and immunomodulating agents). Use of this approach can ultimately lead to increased predictive power relative to directly calculating the commonalities between chemically induced and disease-specific DEGs.

### Biological interpretations of chemical–TF–disease associations to estimate the pivotal TFs involved in efficacies and side effects of CTD chemicals

**Fig. 4:**
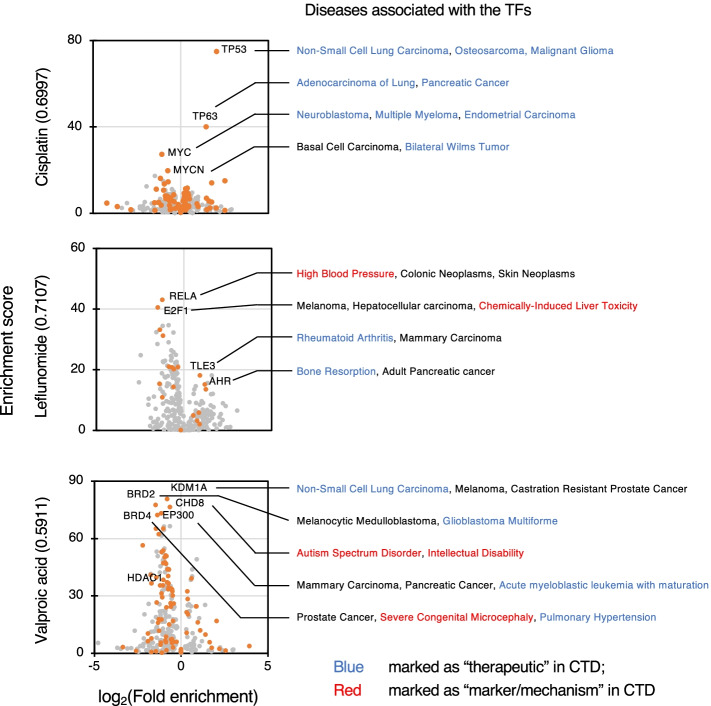
Biological interpretations of the chemical–TF–disease associations. Representative diseases are associated with the predicted target TFs of three chemicals. Dots indicate individual TFs and are colored orange only if they match the chemical–disease associations recorded in CTD database. Chemical–disease associations are indicated in blue (marked as “therapeutic” in CTD) or red (marked as “marker/mechanism” in CTD). AUROC scores showing the accuracy of chemical–TF association inference are shown in parentheses following the names of chemicals

Figure 4 shows representative results of chemical–TF–disease associations constructed using the proposed approach for cisplatin, leflunomide, and valproic acid.

Cisplatin is an antineoplastic chemotherapy agent that acts by crosslinking DNA, resulting in DNA damage; in cancer cells, the drug activates ferroptosis and apoptosis [36–39]. Among the predicted target TFs, tumor suppressors TP53 and TP63 showed the most significant enrichment for genes up-regulated by cisplatin administration [40, 41]. By contrast, oncogenic factors MYC and MYCN were the most enriched TFs for the down-regulated genes [42]. These four TFs were assigned to various types of cancers in DisGeNET, leading to the prediction that cisplatin is associated with neoplasms; this prediction is firmly consistent with the evidence [38, 39]. Although the primary MoA of cisplatin is well known to involve crosslinking of genomic DNA, there is no evidence to support a direct interaction between TP53 or MYCs and cisplatin. Therefore, it is likely that cisplatin indirectly promotes the anti-tumor function of TP53 while suppressing the cancer-inducing function of MYCs. This suggests that our approach is useful for discovering indirect mediators in pharmacological processes that cannot be found by methods based on molecular structures, such as docking simulation and structure-based machine learning.

Leflunomide (LEF) is approved for treating adult rheumatoid arthritis. The drug acts mainly through direct inhibition of dihydroorotate dehydrogenase (DHODH), which is thought to impair proliferation of inflammatory T cells by blocking de novo pyrimidine biosynthesis [43–47]. Controversially, some reports have shown that impairment of cell proliferation by LEF is not rescued by uridine supplementation, implying that the drug also exerts DHODH-independent effects [48, 49]. Remarkably, our ChIPEA-based approach revealed that the most enriched TF for the LEF-induced up-regulated genes was TLE3, which suppresses cellular proliferation by inhibiting MAPK pathways [50, 51]. Another intriguing TF enriched for the up-regulated DEGs was AHR, an important regulator of inflammation in the immune system. This is consistent with the fact that LEF induces the AHR–ARNT interaction, thereby attenuating bone erosion in rheumatoid arthritis [52]. In addition, LEF was also predicted to inactivate the NF-κB pathway component RELA and the key cell cycle promoter E2F1, both of which activate cellular proliferation [53, 54]. These results suggest that LEF suppresses proliferation of inflammatory cells in rheumatoid arthritis patients by influencing those TFs in a manner independent of DHODH. The top hit of RELA, along with the well-known interaction of NF-κB with the immunoglobulin light-chain enhancer in B cells, is consistent with previous findings that LEF prevents immunoglobulin production through inhibition of tyrosine kinase activity [55, 56]. Other than its antirheumatic effect, LEF has potential anticancer activity [57–59]. Although the underlying mechanism is elusive, our ChIPEA suggested that LEF suppresses tumor growth by activating TLE3 and inactivating RELA and E2F1, possibly via the same mechanisms involved in rheumatoid arthritis treatment. Thus, our proposed approach can be used to reveal MoAs that would not be expected from consensus interpretations.

Valproic acid (VPA), a structurally simple fatty acid, has anticonvulsant properties and has been widely applied in the treatment of epilepsy [60]. It is also a potent HDAC inhibitor and is under investigation as a treatment for various cancers [61–64]. In this analysis, several factors involved in chromatin remodeling, such as KDM1A, BRD2/4, and EP300, were significantly enriched to the down-regulated genes in response to VPA administration. Given that aberrant chromatin remodeling is a hallmark of oncogenesis [65], it is reasonable to speculate that chromatin landscapes would be moderately regulated by those factors, and that such effects could be responsible for the anticancer actions of VPA. Furthermore, we detected significant enrichment of the chromatin remodeler CHD8, which genome-wide association studies have shown to be strongly associated with the risk of autism spectrum disorder in humans and in mouse models [66–68]. A well-known adverse effect of VPA is that exposure during pregnancy increases the risk of autism in children [69], although the molecular mechanism underlying this process remains poorly understood. Our results suggest that prenatal use of VPA affects the activity of CHD8, thereby perturbing the target genes involved in neural development. Interestingly, lower CHD8 binding enrichment was observed for the genes perturbed after treatments with the HDAC inhibitor vorinostat (Fig. 2c). This suggests that the potential key role of CHD8 in VPA-induced autism is HDAC-independent, an idea that is also supported by the lack of evidence showing a relationship between maternal use of vorinostat and neonatal autism. These findings demonstrate that our proposed approach has the potential to elucidate the MoAs involved in adverse effects of chemicals, and could therefore identify possible preventive strategies.

## Discussion

In this paper, we present a novel computational approach for elucidating MoAs, focused on pivotal TFs, using large-scale data sets of chemically induced DEGs. This method enables estimation of the efficacies and side effects of given chemicals. In the proposed approach, we identified the TFs that organize the expression of chemically induced DEGs before addressing the associations with diseases based on gene/protein–disease databases. We also tested a method that did not consider key TFs involved in MoAs; in that approach, chemical–disease associations were defined based on the commonalities of chemically induced and disease-specific DEGs. In terms of accuracy, the performance of the proposed approach was superior. This is likely because gene expression changes are the final outcome of complex pharmacophysiological cascades; consequently, direct comparison of chemically induced DEGs with disease-specific DEGs will be influenced by secondary effects and other unknown factors. By contrast, TFs that integrate chemically induced DEGs are likely to be the direct targets of the corresponding compounds, or at least more proximal to them. Indeed, AR and ESR1 were identified as the targets of the sex hormones testosterone and estradiol, respectively, whereas HDACs and BRDs were suppressed upon treatment with the anti-tumor drugs vorinostat and JQ1. In addition, we found that TP53/TP63 and MYCs integratively organized the expression of the up- and down-regulated genes that were responsive to cisplatin administration, respectively, revealing these proteins as pivotal factors in the MoAs of cisplatin’s anti-tumor activities. Thus, in the context of pharmaceutical research, it might be only part of a complex system: ChIPEA is useful for elucidating MoAs from the epigenetic standpoint by revealing pivotal factors within the black box between input (drug administration) and output (gene expression changes). Our proposed method is easily performed using the “Enrichment Analysis” tool from the ChIP-Atlas website (Additional file 1: Fig. S3), where one can identify TFs simply by submitting a list of the DEGs that were identified after drug administration.

Genome-wide identification of TF binding sites based on inference of binding motifs is widely used to understand transcriptional regulation [70]. Previously, a method was proposed to model gene regulatory relationships based on predicted TF binding motifs [71]. On the other hand, we utilized sets of target genes for each TF, which were constructed based on actual ChIP-seq experimental data in a motif-independent manner [27]. Relative to motif-based methods, our approach, which is based on ChIP-seq data, has the advantage of taking into consideration the actual TF binding sites within a specific cellular state. It is important to note that TFs do not always bind to specific binding motifs, even those that are statistically well-defined. Similarly, it is not uncommon for a TF to bind to sites that totally differ from its modeled motifs. This observation may partially explain the undesirable results that are obtained when using a DNA sequence motif-based method to estimate chemical–TF associations for chemically induced genes (Additional file 1: Fig. S4) [72], though these motif-based methods are of value when ChIP-seq data for specific TFs cannot be obtained from publicly available resources.

To predict chemical–target interactions, chemogenomics methods have been developed based on the compound molecular structures and protein sequence motifs or structural features [4–8]. When aiming to find proteins that directly interact with given ligands, these methods are generally efficient, with high prediction accuracy as long as the structures of the proteins and compounds have been well characterized. As a complementary approach, our method is applicable to proteins and compounds with less defined structures, as we do not focus primarily on direct drug–target interactions. Our approach only requires transcriptome analysis data for given chemicals, and is therefore capable of providing novel insights into chemical–protein associations fully based on biological experiments and public ChIP-seq data. In recent years, supervised machine learning algorithms have been used to predict drug targets and novel indications [9, 10]. Predictions made using this kind of approach can be very accurate if the system is provided with sufficient unbiased knowledge about specific drugs, proteins, and diseases. Such machine learning approaches will become more powerful when combined with TF-based knowledge obtained from our proposed method, which will allow gene-regulatory networks based on actual ChIP-seq experiments to contribute more accurate and explainable predictions.

Although the ChIPEA procedure itself is not novel, we show in this paper that the proposed ChIPEA-based approach is capable of identifying key regulators that are the direct targets of, or are primarily involved in the MoAs of, given bioactive compounds, implying that it could be used to make important contributions to the pharmaceutical field. For instance, ChIPEA could be used to analyze data from high-throughput expression screening of thousands of chemicals. The conventional approaches for screening candidate compounds include Gene Ontology and pathway enrichment analyses, which can be used to identify common features among chemically induced genes. In addition, our proposed approach provides insight into the regulatory mechanism acting upstream of these genes, allowing identification of drug candidates targeting the desired TFs. Furthermore, our method could also be applied to the transcriptome data of unapproved drugs, including compounds under development and those that failed to be approved in clinical trials. Identification of TFs primarily involved in MoAs, together with the factors associated with potential side effects, could shed light on the potential utility of repositories of compounds hoarded in pharmaceutical industries. In addition, our method could be used to analyze approved drugs, including those with more or less well-defined MoAs. Other possible applications include drugs composed of unidentified ingredients, such as traditional herbal medicines. Potential therapeutic effects, side effects, and novel targets and actions can be inferred by identifying TFs involved in unexpected physiological pathways.

Finally, we wish to discuss the limitations and the extensibility of the proposed ChIPEA-based method. In this study, we considered only TF binding sites adjacent to the chemically induced genes (target range: transcription start site ± 5 kb). In general, genes are regulated by a complex series of enhancers at a short or long distance, of which a fair proportion are considered to fall outside the target range of ChIPEA. Thus, it would be informative to use chromatin accessibility data (DNase-seq and ATAC-seq) to analyze TF binding at longer ranges from chemically perturbed genes. Furthermore, genome-wide chromatin conformation capture (Hi-C) data is most suited to identifying chemically perturbed genes and the long-range TF binding sites that are sometimes observed for cis-regulatory elements. If ATAC-seq data are available for a broad coverage of chemicals, a more direct type of input data for ChIPEA would be the open chromatin regions, rather than the genes, rearranged by chemical administration. In our proposed method, the gene–TF matrix needs to be binarily abstracted as “binding” or “non-binding”, which does not take into account the broad range of binding affinities observed. Development of a weighted enrichment analysis method that includes an algorithm to factor in the binding affinity between each TF and specific gene loci, such as the statistical values obtained using the MACS2 peak calling procedure (*q*-values), and the distance between genes and TF binding sites [73] would address this issue. It should be kept in mind that ChIPEA focuses on the “binding” patterns of TFs to chemically induced genes; however, “binding” does not necessarily mean that there is a regulatory relationship. It would also be best to experimentally validate the predicted results for pivotal TFs in order to determine their relationships within regulatory networks. Finally, because we are using experimental ChIP-seq data, TFs lacking public ChIP-seq data cannot be analyzed using ChIPEA. However, because the number of ChIP-seq experiments is steadily increasing, the ChIPEA-based approach will become increasingly powerful in the future.

## Conclusions

In this paper, we introduced a computational approach to elucidating the epigenetic landscape of drug responses, in which large-scale public ChIP-seq experiment data were analyzed to identify key TFs acting upstream of chemically induced genes. Chemical–TF associations were predicted by ChIPEA of chemically induced expression profiles and validated using a chemical–protein association database. Furthermore, chemical–TF–disease associations were constructed by linking the TFs with known disease-associated proteins. Together, our findings demonstrate that ChIPEA using public ChIP-seq data is an efficient way to identify master regulators involved in MoAs from an epigenetic perspective. Therefore, this approach is a powerful means of predicting chemical–TF and chemical–disease associations in a biologically interpretable manner, outperforming methods that do not consider information about the TFs involved in MoAs. Our proposed approach could be used to further understand the MoAs of candidate drugs, as well as to discover unexpected therapeutic effects and side effects of approved drugs.

## Methods

### Datasets

#### Chemically perturbed DEGs from CTD

Gene symbols of genes up- and down-regulated by environmental chemicals and medical drugs were obtained from CTD (chemical–gene interaction; download link, http://ctdbase.org/reports/CTD_chem_gene_ixns.csv.gz; downloaded on May 15th, 2020). CTD is a community-supported genomic resource that provides manually annotated associations among chemicals, genes/proteins, and diseases [20]. In the chemical–gene interaction database, each chemical–gene interaction is addressed in a declarative statement and qualified by a degree: increases, decreases, affects, or does not affect. We used only gene expression profiles of human cells in response to chemical administration with the interaction type “*C* (analog) results in increased/decreased expression of *G* mRNA/protein”, where *C* and *G* represent a chemical and a gene, respectively. The number of DEGs in response to treatment with each chemical varied widely, from 1 to over 6,000. We extracted expression profiles with more than ten each of up- and down-regulated DEGs, yielding a total of 890 gene expression profiles related to 434 chemicals (Additional file 2: Table S1).

#### Genome-wide TF binding experimental data from ChIP-Atlas

We obtained information about genome-wide TF binding sites from ChIP-Atlas, an integrative database that covers almost all public ChIP-seq data submitted to the NCBI SRA [27]. The metadata of all experiments, such as names of antigens and cellular states, are manually curated according to commonly or officially adopted nomenclature. The sequence data are processed with a unified pipeline in which sequenced reads are aligned to a reference genome with Bowtie2 and subjected to peak calling with MACS2. We retrieved full sequencing data from 13,558 experiments (Additional file 2: Table S2) that identified 170,067,307 binding sites (peaks were called with MACS2; *q*-value < 1 × 10^−10^) of 997 TFs in the human genome (download link, http://dbarchive.biosciencedbc.jp/kyushu-u/hg19/allPeaks_light/allPeaks_light.hg19.05.bed.gz; genome version, hg19; downloaded on May 15th, 2020). It is worth noting that all of the binding data in ChIP-Atlas were determined experimentally and therefore are not binding-motif dependent.

#### Gene/protein–disease association

Gene/protein–disease association data were acquired from DisGeNET (version, v7.0; download link, https://www.disgenet.org/static/disgenet_ap1/files/downloads/curated_gene_disease_associations.tsv.gz; downloaded on June 4th, 2020), a large collection of genes and variants associated with human diseases [28]. DisGeNET integrates data from expert-curated repositories and the scientific literature. We retrieved only manually curated data labeled with referenced PubMed IDs, yielding 77,524 gene/protein–disease associations involving 9,334 genes/proteins and 7,687 diseases.

#### Disease-specific DEGs from CREEDS

Disease-specific gene expression profiles were constructed based on gene expression profiles in CREEDS (manual disease signatures v1.0; download link, http://amp.pharm.mssm.edu/CREEDS/download/disease_signatures-v1.0.json; download on June 16th, 2020), a crowdsourcing project aimed at annotating and reanalyzing a large number of gene expression profiles from Gene Expression Omnibus [35]. The gene expression profiles were associated with scores calculated using the characteristic direction method, in which gene expression levels in diseased tissues were compared with healthy controls. Genes with positive or negative expression scores were considered to be up- or down-regulated DEGs, respectively, yielding 554 gene expression profiles associated with 235 diseases.

#### Standard chemical–protein interactome

Compound–protein interaction data were acquired from KEGG DRUG (download link, ftp://ftp.biosciencedbc.jp/archive/kegg-medicus/LATEST/kegg_medicus_drug.csv.zip; downloaded on July 1st, 2019), a comprehensive collection of approved drugs and their target information, including 11,550 chemical–protein interactions involving 1,458 chemicals and 768 proteins [29].

#### Standard chemical–disease associations

Chemical–disease association data were acquired from CTD (chemical–disease interactions; download link, http://ctdbase.org/reports/CTD_chemicals_diseases.tsv.gz; downloaded on June 15th, 2020). In total, we obtained 219,317 chemical–disease associations involving 9,855 chemicals and 3,244 diseases, curated from 78,582 papers.

### The proposed ChIPEA-based approach

#### Identification of key TFs that organize the expression of chemically induced genes using ChIPEA

Taking full advantage of this enormous quantity of data, we performed enrichment analysis termed ChIPEA to profile TFs whose binding sites were enriched around chemically induced genes of interest. In particular, starting with the gene symbols of up- and down-regulated genes induced by a query chemical, we counted the overlaps between the transcription start site ± 5 kb regions of chemically induced DEGs and peak-call data of all TF-related experiments archived in ChIP-Atlas, using the “intersect” command of BEDTools2 (version, v2.23.0) [74]. Enrichment scores (−log_10_[*p*-values]) were calculated using the two-tailed Fisher’s exact probability test, with the null hypothesis that the two data sets (up- and down-regulated genes) overlap with the ChIP-seq peak-call data in the same proportion; fold enrichment values were returned at the same time. If a chemical–TF association was given by multiple ChIP-seq experiments, the highest enrichment score was adopted. This ChIPEA procedure was proposed previously [27].

#### Relating the chemical–TF matrix identified using ChIPEA to TF–disease associations

Data for gene/protein–disease associations derived from DisGeNET (formula 1; where P_*m*_ and D_*n*_ represent a protein and a disease, respectively) were correlated with the chemical–TF associations determined using ChIPEA (formula 2; where C*i*, T*j*, and E_*ij*_ represent a chemical, a TF, and an enrichment score, respectively) when T_*j*_ was also included in DisGeNET as P_*m*_ (formula 3). The enrichment scores calculated by ChIPEA were also used to evaluate the probability of each chemical–disease prediction (formula 4).

1 $${\text{P}}_{m} {-}{\text{D}}_{n}$$

2 $${\text{C}}_{i} {-}{\text{T}}_{j} {-}{\text{E}}_{ij}$$

3 $${\text{T}}_{j} = {\text{P}}_{m}$$

4 $${\text{C}}_{i} {-}{\text{T}}_{j} {-}{\text{D}}_{n} {-}{\text{E}}_{ij}$$

If a chemical–disease pair was predicted via multiple TFs, the highest enrichment score was adopted.

#### Calculation of global AUROC and AUPR

After evaluating their validity, all predicted chemical–TF or chemical–disease associations were arranged into a single *m* × *n* matrix consisting of enrichment scores with correctness information, where *m* was the total number of chemicals and *n* was the number of TFs or diseases, respectively. We then stored the maximum value of enrichment scores within each column (TF or disease) into a vector with *n* elements. We generated ROC and PR curves and summarized the results into global AUROC and AUPR scores as described in the Results section.

### Baseline methods

#### Motif-based enrichment analysis using the regulatory genomics toolbox [72]

In the motif matching step, a set of TF motifs identified from several main repositories was compared to the genomic regions of chemically induced genes without taking into account the DNase-seq signals. Subsequently, motif enrichment analysis was performed for each chemical (download link, http://www.regulatory-genomics.org/wp-content/uploads/2017/03/RGT_MotifAnalysis_FullSiteTest.tar.gz; downloaded on July 12th, 2021). In particular, Fisher’s exact test was used in order to determine whether the chemically induced genes were enriched for particular TFs. Enrichment scores (−log_10_[*p*-values]) were calculated using the two-tailed Fisher’s exact probability test, with the null hypothesis that the two data sets (up- and down-regulated genes) overlap with the TF motif data in the same proportion; fold enrichment values were returned at the same time. Testosterone-, vorinostat-, and JQ1-induced genes were obtained from CTD database.

#### DEG-connected method for predicting chemical–disease associations

Chemically induced and disease-specific expression changes in 28,268 genes were classified as up-regulated, down-regulated, or non-DEG; RefSeq genes were obtained from the UCSC genome annotation database of the human genome (download link, http://hgdownload.soe.ucsc.edu/goldenPath/hg19/database/refFlat.txt.gz; genome, hg19; downloaded on July 7th, 2020). All chemical and disease profiles were further arranged into two-by-two cross tabulations for each of the chemical–disease pairs in two ways (“DEGs vs. non-DEGs” and “up- vs. down-regulated genes”), as illustrated in Table 1.

**Table 1:** Cross tabulation showing the frequency distribution of chemically induced and disease-specific genes, with C and D representing a chemical and a disease, respectively

		Disease ***D***				Disease ***D***	
		DEGs	non-DEGs			Up	Down
Chemical *C*	DEGs	*n1*	*n2*	Chemical *C*	Up	*n1*	*n2*
	non-DEGs	*n3*	*n4*		Down	*n3*	*n4*
	**“DEGs vs. non-DEGs” comparison**				**“up- vs. down-regulated genes” comparison**		

The chemical–disease associations were evaluated based on *p*-values calculated using the two-tailed Fisher’s exact probability test with the null hypothesis that the comparative gene expression patterns in response to a given *C* (chemical) and *D* (disease) (*n1*, *n2*, *n3*, and *n4*) were uniformly distributed. Chemical–disease pairs with smaller *p*-values were considered to be more firmly associated.

## Supplementary Information

**Additional file 1: Fig. S1 Comparison of gene expression profiles and annotated proteins contained in different databases. a, c** Venn diagram showing genes shared between the chemical–gene (CTD and L1000) and disease–gene (CREEDS) association databases (a), and proteins shared between the ChIP-seq experiment (ChIP-Atlas) and gene/protein–disease association (DisGeNET) databases (c). **b, d** Bar charts showing the frequencies with which the genes appeared in CTD and CREEDS, with L1000 genes colored in orange (CTD) and green (CREEDS) (b), and the frequencies with which proteins were defined as disease-associated proteins in DisGeNET, with ChIP-Atlas TFs colored in blue (d). **Fig. S2 Factors potentially affecting the distribution of AUROC scores.** AUROC scores, sorted according to the highest enrichment score for each (**a, c**) chemical and (**b, d**) the number of DEGs that were used to predict (**a, b**) chemical–TF and (**c, d**) chemical–disease associations using the proposed ChIPEA-based approach. **Fig. S3 Visual manual for GUI-based ChIPEA. a.** Submission form for ChIPEA on the website. GUI-based ChIPEA is provided on the ChIP-Atlas website (termed “Enrichment Analysis” tool; https://chip-atlas.org/enrichment_analysis). When used to identify pivotal TFs involved in drug MoAs, genome assembly should be set as “hg19/hg38” (hg19 was used in this paper). “TFs and others” needs to be selected in panel “1. Antigen Class”. “2. Cell type Class” and “3. Threshold for Significance” may be changed by the user according to demand. The “4. Enter dataset A” dialog box is to be filled in with the list of up-regulated genes, and the box “5. Enter dataset B” is for down-regulated genes. After specifying the “Distance range from TSS” in the “6. Analysis description” panel, the user can press the “Submit” button to submit the parameters to the server, and ChIPEA will initialize immediately. **b.** Interpretation of the results. The overlaps between the genomic loci (originating from panels 4 and 5 of the submission form) and reference peak call data (specified on upper panels 1–3 of the submission form) are counted using the bedtools intersect command (BedTools2; ver 2.23.0). The results are returned in html and tsv format. *p*-values are calculated using a two-tailed Fisher’s exact probability test. The null hypothesis is that the intersection between the reference peaks and the data submitted in panel 4 occurs at the same proportion as for the data in panel 5 of the submission form. *q*-values are calculated using the Benjamini & Hochberg method. Fold enrichment was calculated by dividing the result from column 6 by the result from column 7 for a given row of data. If the ratio > 1, the rightmost column is “TRUE”, meaning that the protein from column 3 is more likely to bind to the variable from panel 4 than to that from panel 5. **Fig. S4 Identification of target TFs using a motif analysis-based method.** TF binding profiles of three chemicals predicted by motif analysis using DEGs from CTD (testosterone, vorinostat, and JQ1). Dots indicate individual TFs and are colored orange only if they match the chemical–target associations recorded in KEGG DRUG database. AUROC scores are shown in the upper right corner of the volcano plot of each chemical.

**Additional file 2: Table S1. List of CTD chemicals.** Column 1, CTD ID; column 2, chemical name. **Table S2. List of ChIP-seq experiments.** Column 1,  Experiment ID; column 2, ChIP-seq antigen. **Table S3. Predicted chemical–TF associations using ChIPEA for Fig. 2c.** **a** Testosterone; **b** Estradiol; **c** Tamibarotene; **d** Vorinostat; **e** JQ1 compound.

## Data Availability

We used freely available datasets, and the identifiers of the datasets are provided in Table S1 and S2 in Additional file 2; specific version numbers or dates of access/download are indicated in the Methods section. The code related to the proposed approach is available free of charge via the Internet at https://github.com/zouzhaonan/moa_tfea/.

## Abbreviations

AUPR: Area under the precision-recall curve
AR: Androgen receptor
AUROC: Area under the receiver operating characteristic curve
ChIPEA: Chromatin immunoprecipitation sequencing-based enrichment analysis
ChIP-seq: Chromatin immunoprecipitation sequencing
CMap: Connectivity Map
CREEDS: Crowd Extracted Expression of Differential Signatures
CTD: Comparative Toxicogenomics Database
DEGs: Differentially expressed genes
DHODH: Dihydroorotate dehydrogenase
ES: Enrichment score
ESR1: Estrogen receptor 1
ESR2: Estrogen receptor 2
KEGG DRUG: Kyoto Encyclopedia of Genes and Genomes DRUG database
LINCS: National Institute of Health Library of Integrated Network-Based Cellular Signatures
MoA: Mode of action
PHTS: Phenotype-based high-throughput screening
PR: Precision-recall
RARA: Retinoic acid receptor alpha
ROC: Receiver operating characteristic
TF: Transcription factor
TG-GATEs: Toxicogenomics Project-Genomics Assisted Toxicity Evaluation system
VPA: Valproic acid
