# Supplementary material for: Epigenetic landscape of drug responses revealed through large-scale ChIP-seq data analyses
Source: BMC Bioinformatics. 2022 Jan 24;23:51. doi: 10.1186/s12859-022-04571-8 (PMC8785570; doi:10.1186/s12859-022-04571-8)
Supplement: Supplementary file 1 — Additional file 1: Fig. S1 Comparison of gene expression profiles and annotated proteins contained in different databases. a, c Venn diagram showing genes shared between the chemical–gene (CTD and L1000) and disease–gene (CREEDS) association databases (a), and proteins shared between the ChIP-seq experiment (ChIP-Atlas) and gene/protein–disease association (DisGeNET) databases (c). b, d Bar charts showing the frequencies with which the genes appeared in CTD and CREEDS, with L1000 genes colored in orange (CTD) and green (CREEDS) (b), and the frequencies with which proteins were defined as disease-associated proteins in DisGeNET, with ChIP-Atlas TFs colored in blue (d). Fig. S2 Factors potentially affecting the distribution of AUROC scores. AUROC scores, sorted according to the highest enrichment score for each (a, c) chemical and (b, d) the number of DEGs that were used to predict (a, b) chemical–TF and (c, d) chemical–disease associations using the proposed ChIPEA-based approach. Fig. S3 Visual manual for GUI-based ChIPEA. a. Submission form for ChIPEA on the website. GUI-based ChIPEA is provided on the ChIP-Atlas website (termed “Enrichment Analysis” tool; https://chip-atlas.org/enrichment_analysis). When used to identify pivotal TFs involved in drug MoAs, genome assembly should be set as “hg19/hg38” (hg19 was used in this paper). “TFs and others” needs to be selected in panel “1. Antigen Class”. “2. Cell type Class” and “3. Threshold for Significance” may be changed by the user according to demand. The “4. Enter dataset A” dialog box is to be filled in with the list of up-regulated genes, and the box “5. Enter dataset B” is for down-regulated genes. After specifying the “Distance range from TSS” in the “6. Analysis description” panel, the user can press the “Submit” button to submit the parameters to the server, and ChIPEA will initialize immediately. b. Interpretation of the results. The overlaps between the genomic loci (originating from panels 4 and 5 of the su [file 12859_2022_4571_MOESM1_ESM.pdf]

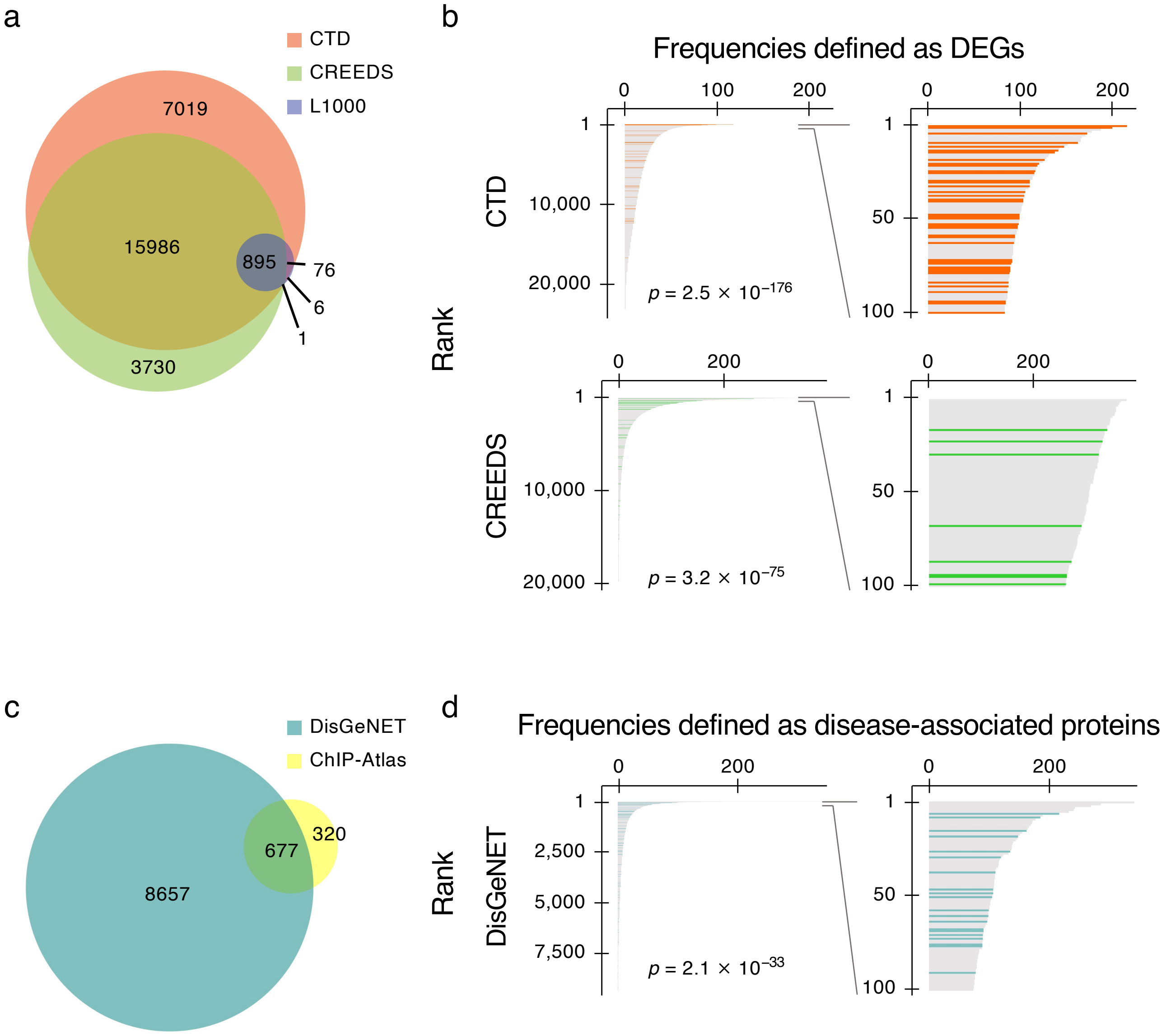

Additional file 1: Figure S1

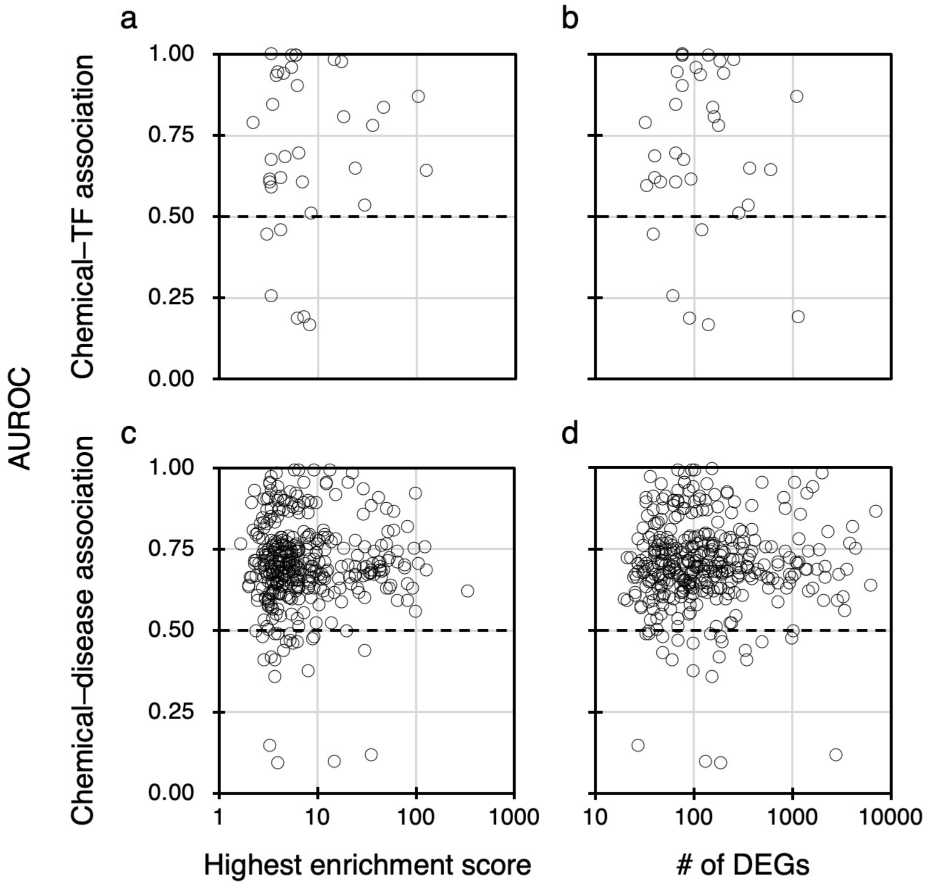

Additional file 1: Figure S2

a

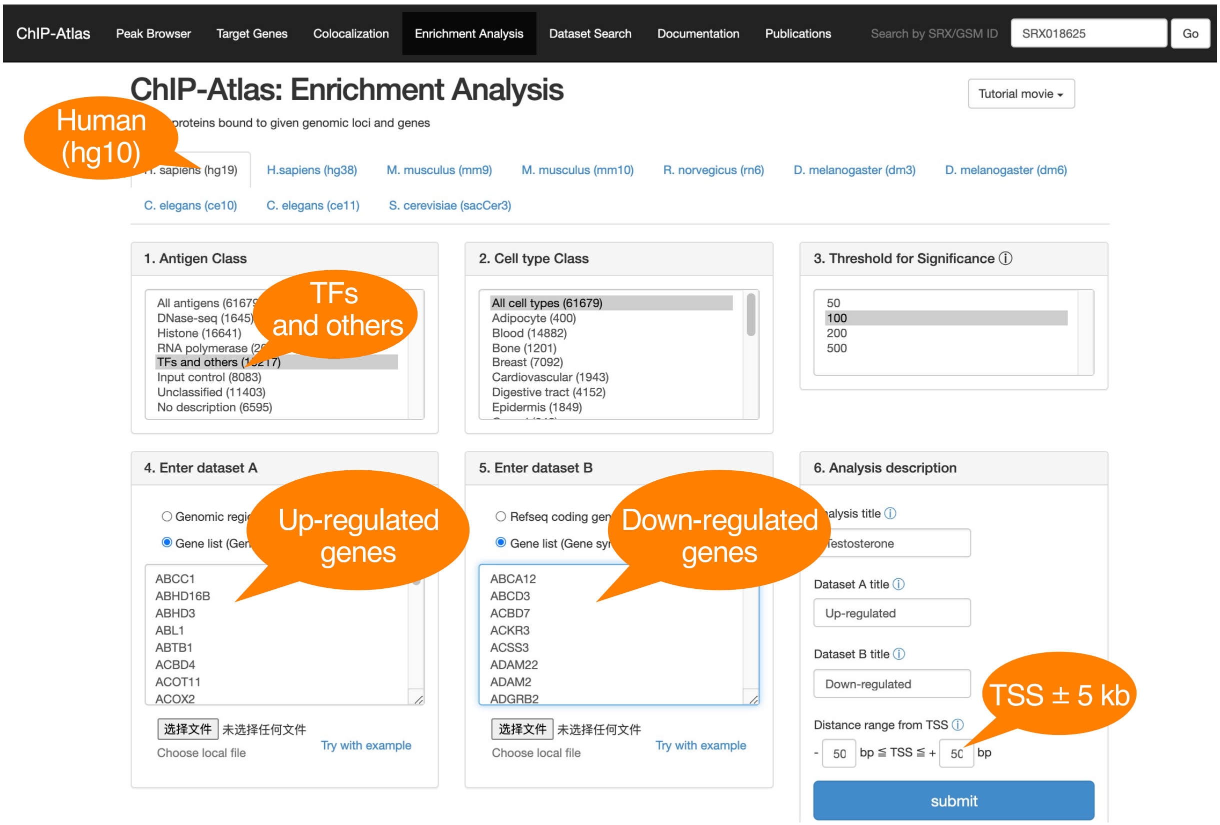

b

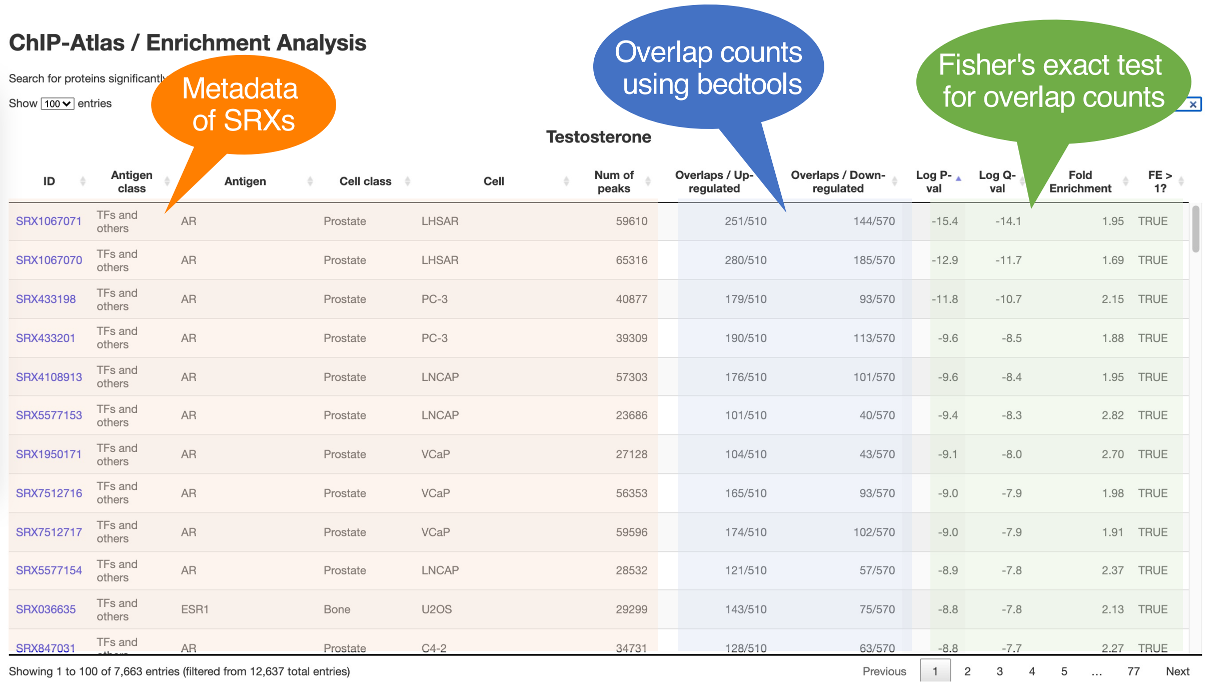

ID	Antigen class	Antigen	Cell class	Cell	Num of peaks	Overlaps / Up-regulated	Overlaps / Down-regulated	Log P-val	Log Q-val	Fold Enrichment	FE > 1?
SRX1067071	TFs and others	AR	Prostate	LHSAR	59610	251/510	144/570	-15.4	-14.1	1.95	TRUE
SRX1067070	TFs and others	AR	Prostate	LHSAR	65316	280/510	185/570	-12.9	-11.7	1.69	TRUE
SRX433198	TFs and others	AR	Prostate	PC-3	40877	179/510	93/570	-11.8	-10.7	2.15	TRUE
SRX433201	TFs and others	AR	Prostate	PC-3	39309	190/510	113/570	-9.6	-8.5	1.88	TRUE
SRX4108913	TFs and others	AR	Prostate	LNCAP	57303	176/510	101/570	-9.6	-8.4	1.95	TRUE
SRX5577153	TFs and others	AR	Prostate	LNCAP	23686	101/510	40/570	-9.4	-8.3	2.82	TRUE
SRX1950171	TFs and others	AR	Prostate	VCaP	27128	104/510	43/570	-9.1	-8.0	2.70	TRUE
SRX7512716	TFs and others	AR	Prostate	VCaP	56353	165/510	93/570	-9.0	-7.9	1.98	TRUE
SRX7512717	TFs and others	AR	Prostate	VCaP	59596	174/510	102/570	-9.0	-7.9	1.91	TRUE
SRX5577154	TFs and others	AR	Prostate	LNCAP	28532	121/510	57/570	-8.9	-7.8	2.37	TRUE
SRX036635	TFs and others	ESR1	Bone	U2OS	29299	143/510	75/570	-8.8	-7.8	2.13	TRUE
SRX847031	TFs and others	AR	Prostate	C4-2	34731	128/510	63/570	-8.8	-7.7	2.27	TRUE

Showing 1 to 100 of 7,663 entries (filtered from 12,637 total entries) Previous 1 2 3 4 5 … 77 Next

Additional file 1: Figure S3

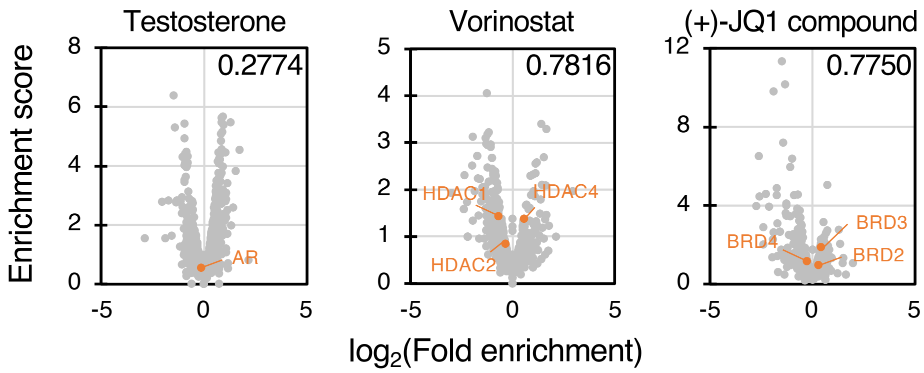

Additional file 1: Figure S4
